# Whose Truth Is Ground Truth?: Consequences of Label Choice on Machine Learning Models Predicting Depression

**DOI:** 10.21203/rs.3.rs-9488927/v1

**Published:** 2026-07-30

**Authors:** Natasha Tonge, Leah Adams

**Affiliations:** George Mason University; George Mason University

**Keywords:** depression, electronic health record, measurement-based care, machine learning, AI

## Abstract

Machine learning (ML) models developed using electronic health record (EHR) data frequently rely on provider-documented diagnoses as ground truth to develop models, despite substantial variation in how mental health conditions are diagnosed and recorded. To empirically demonstrate the impact of diagnostic label source, we examined how use of patient-reported, provider-coded, or patient–provider concordant depression labels impact the performance and feature importance of tree-based ML models.

We analyzed EHR data from 644,387 adults (2012–2019) in the OCHIN ADVANCE network. Depression outcomes were defined using (1) patient-reported PHQ-9 scores, (2) provider-coded ICD diagnoses, and (3) concordance. Predictors included demographics, vital signs, chronic conditions, and social determinants of health. Classification and regression trees (CART) were trained using 70/30 splits and 10-fold cross-validation. Performance was assessed using sensitivity, specificity, AUC, and F1 score. SHapley Additive exPlanations (SHAP) quantified feature importance.

Model performance was modest (AUC = .62–.64); sensitivity was highest for provider-indicated depression (0.33). Feature importance differed substantially by labeling method: anxiety was the only consistently influential predictor across label strategies. Demographic features were highly influential in provider-labeled models but less important in patient-reported or concordant label strategies.

We empirically demonstrated how depression label choice alters ML model performance and feature interpretation. Higher AUC values from provider-derived labels were offset by amplified demographic feature importance, raising concerns about the interaction of label choice and bias. Our findings underscore the importance of transparent reporting, comparing labeling strategies, and including patient-reported outcomes in model development to support equitable mental health AI.

## Introduction

Machine learning (ML) models are statistical applications that work especially well for gaining predictive insight into large, complex datasets (Beam & Kohane, 2018). As a result, these models and techniques have been applied to “big data” such as the richly featured administrative and diagnostic data present in electronic health records (EHR) in the hopes of uncovering meaningful patterns that can improve patient diagnosis or prognosis (Chen et al., 2019). EHR data is appealing for ML applications because of the volume of data collected from routine clinic operations (e.g., appointment dates and times, types of clinics), diagnostic tests (e.g., medical imaging, self-report questionnaires), and billing information (e.g., diagnoses, treatment, medications). For example, in areas such as radiology that depend heavily on medical imaging technology to identify physical anomalies as part of the diagnostic process, ML models can be beneficial for everything from improving image quality to assisting clinicians with the diagnostic process itself in the form of clinical decision support (Choy et al., 2018).

Applications to mental health are less widespread. Within mental health, suicide risk prediction is one of the few areas where ML models are actively being developed and tested. The Department of Veterans’ Affairs’ REACHVET program was developed using EHR to create an ML model that has been deployed nationally to identify veterans at high risk of suicide (Matarazzo et al., 2023). REACHVET is a fairly unique program in terms of scale and application of ML in the domain of mental healthcare. The success in the VA when utilizing these methodologies to help detect disease, coordinate care and overall enhance patient outcomes underscores a need for continued development of new ML-based algorithms for behavioral healthcare applications. As interest in these models accelerates, it is essential to evaluate how other mental health conditions can be reliably identified or predicted using complex or “big data” datasets.

### Depression is a Desirable but Challenging Target for Predictive Machine Learning Models

Depression represents a salient target for future intervention and algorithm development. Mortality and morbidity are high and within hospital settings, depression can easily go undiagnosed or unrecognized amid comorbid health conditions (Benton et al., 2007; Carney & Freedland, 2003; Cuijpers & Schoevers, 2004). Yet depression has several unique features ranging from how data on depression are collected and recorded in the electronic health record (EHR) to how the diagnosis is made. These features create key points of departure from conditions that have been successfully and reliably been subjects of study with ML models in healthcare systems. Understanding how these unique features influence ML models of depression should be a first step in algorithm development but is easily overlooked.

Only a subset of researchers have looked to apply ML prediction models to depression and frequently use EHR data (Bhadra & Kumar, 2022; Linthicum et al., 2019). However, unlike suicide, which is inferable in an EHR through combinations of records related to reasons for death or radiology where diagnostic tests rely on presence of visual information, the diagnosis of depression is complicated by heterogeneity of symptoms and a range of approaches used to assess those symptoms. Further, assessment of depression is predicated on patient report of symptoms, which are commonly obtained through discussion with the provider.

Diagnosing mental health challenges is comparatively made more difficult by the subjectivity of symptoms and necessity of self- or informant-report. Compared to cancer, death by suicide requires either that the intent of the patient be known or status is assumed based on observable characteristics (e.g., self-inflicted injury of unknown intent; (Bakst et al., 2016) at time of death. Depression is even more subjective. Depression diagnosis can start with the patient’s perception that they are feeling depressed, sad, or down, their experiences of other symptoms, perception of functional impairment, and memory of the duration and persistence of symptoms (American Psychiatric Association, 2022). The patient must be asked about their internal psychological state and about patterns of their own behavior or they can report these symptoms via questionnaires. Clinical observations may be used, but the patient’s report is interpreted by the physician in order to render the diagnosis. If using the ICD-10, billable conditions need only be recognized by a qualified healthcare provider and a diagnosis can be assigned based on clinician perspective alone. However, in the absence of diagnostic tests for depression, diagnosis depends on patient-provider communication and collaboration in the diagnostic process. In the absence of efficient patient-provider communication around symptoms, recorded EHR diagnoses can be biased, which in turn can bias labels used for supervised machine learning.

Despite the complexity inherent in identifying and correctly diagnosing depression from EHR records, most studies using machine learning rely exclusively on codes or notes rendered by the physician. In a recent review by Nickson et al. (2023), 19 studies using EHR data to predict and diagnose depression using machine learning methods were identified. Of those 19 studies, only one made use of self-report data to identify depression diagnosis. Using the Hospital Anxiety and Depression Scale (HADS; Zigmond & Snaith, 1983), demographic features, and random forest models, authors Sau and Bhakta (2017) were able to train a random forest model to classify around 500 geriatric patients who had been diagnosed with anxiety/depression using the HADS in Kolkata, India with 89% accuracy. They identified demographic features, diabetes, hypertension, hearing or visual impairment, and recent bereavement as the most important predictors for predicting depression or anxiety diagnosis.

The majority of studies relied on billing codes, presence of an anti-depressant prescription, or both. None of the studies surveyed used a self-reported or other patient measure in conjunction with diagnostic codes rendered by the provider to validate the depression diagnosis the ML models were trained on. Although entries in the medical record that capture diagnosis, such as billing codes and records of prescription medications, tend to agree with each other (Trinh et al., 2011), physician assessment of symptoms and patient report have high levels of disagreement. In one study of over 2,000 patients in primary care clinics, patients scoring high on the Patient Health Questionnaire-9 (PHQ-9; Kroenke et al., 2001), a self-report measure of depressive symptoms, were only diagnosed as depressed 37.7% of the time by their primary care physicians. At the symptom level, Cohen’s Kappa suggested only slight agreement between patients and physicians (0.001–0.101; Ani et al., 2008).

Mismatch between patients’ experiences of mental health problems and providers’ assessments may be particularly pronounced among racially and ethnically minoritized patients, potentially exacerbating existing mental health disparities. Retrospective analyses of physician office visits indicate that Black patients are less likely than White patients to have mental health concerns diagnosed or addressed during a visit, and these differences exceed what would be expected based on underlying prevalence alone (Rotenstein et al., 2023; Stockdale et al., 2008). Similar under-recognition and under-documentation of depressive symptoms have been found among Chinese and Latinx patients in California (Garcia et al., 2021). Complementing these observational findings, Adams and colleagues’ (2014) experimental work using simulated patients with probable depression demonstrates the risks of relying on physician reports, as physicians prioritized physical symptoms and expressed lower diagnostic certainty when evaluating a Black patient when compared to an otherwise identical White patient. Beyond under diagnosis, cultural differences in mental health symptom expression may lead to *mis*diagnosis more broadly, further complicating the use of diagnostic labels in ML models.

## Machine Learning Methods and Diagnostic Labeling Challenges

To understand why these diagnostic complexities are relevant to algorithm development, it is useful to briefly review how machine learning models learn from data and what kinds of inputs they require to make predictions. ML models have several ways of “learning” but two of the most popular are supervised and unsupervised learning. In unsupervised learning, the algorithms optimize patterns in the data so that variables or features that are most closely related to each other are grouped. The researcher then observes the groupings to make inferences. In supervised learning, the researcher must start with data that informs the model specifications to make predictions. This data, known as training data, must be labeled (e.g., as with positive or negative for a disease) and then the resulting model may be applied to new, unseen data. There are also more sophisticated approaches such as deep neural networks, semi-supervised learning, and reinforcement learning; however, these two foundational learning types have been widely used to create predictive, diagnostic, and prognostic models.

For supervised learning, this means that a disease state must be knowable and labeled appropriately or else the model can “learn” incorrect or biased predictions. In the case of cancer, there is a wealth of information that can be clearly labeled to train a model. A modern cancer diagnosis can incorporate family history and clinical interview for screening, biopsy and other lab tests, and medical imaging (*Tests and Procedures Used to Diagnose Cancer - NCI*, 2015), which can all be read into the medical record. When imaging is used, it enables the physician to locate the cancerous growth and determine its size. That imaging information can be saved in the medical record and, when recorded outcomes such as whether the growth is cancerous or not, tests used to confirm or further describe the diagnosis (e.g., biopsy, staging), and the prescribed treatments (e.g., radiation, chemotherapy, surgery) are combined in training data, there is a reliable and valid set of information about whether the patient had cancer. Ultimately, patient or informant report of symptoms that can be associated with cancer progression may help with screening or predictive or prognostic models, but carry little weight in the diagnostic model relative to objective factors. These qualities of the models highlight the difference in approach for building an ML model to detect depression as opposed to radiology and suicide, we can consider the processes for obtaining and validating the data and how that data appears in the clinical record (Chen et al., 2019).

## The Current Study

Primary care clinicians, who manage the largest proportion of medically complex patients with co-occurring physical and mental health conditions (Moore et al., 2016), face substantial challenges in recognizing depression amid competing demands. This makes primary care settings prime targets for ML-aided clinical decision support systems, which are meant to aid clinicians operating with complex care decisions with diagnostic suggestions, treatment recommendations, or even additional assessment (Sutton et al., 2020); however, most research efforts have focused on secondary-tertiary care with treatment specialists (Susanto et al., 2023). If ML-assisted decision tools are to support this work, we must first understand how different approaches to labeling depression in EHR data shape the models built on those labels. Identifying depression co-occurring with chronic health conditions such as hypertension, hyperlipidemia, and diabetes could be especially impactful if integrated into an EHR to support primary care providers. However, the promise of an outcome that can critically improve care belies a foundational question: Which depression label should ML models learn from? Labels for depression may originate from the patient (self-identified), the treating clinician (physician-identified), or identified by concordance. Because the depression label is what supervised models learn from, the label source is a precursor to the development of scalable, trustworthy, and unbiased ML models (Liang et al., 2022; Teno, 2023) and it is important to understand how the decision to use patient-identified, provider-identified, or patient-provider concordant indications of the depression in ML models impacts model performance and interpretation.

In the current study, we examine data from a large electronic health record with both self-reported depression and EHR-derived depression codes. Our primary aim was to examine the impact of three ways of identifying depression on machine learning models’ AUC: determined by the patient (self-reported symptoms without a corresponding entry in the medical record), determined by the provider (provider entry in the medical record but low symptom endorsement by patient), and patient-provider agreement. We predicted that there would be notable differences between ML models depending on the source of depression diagnostic information. Our second aim was to identify changes to feature, or variable, importance when the means of identifying depression differed. We similarly predicted differences in the three models in feature importance.

## Method

### Data Source

The data for our retrospective cross-sectional study was provided by the Accelerating Data Value Across a National Community Health Center Network (ADVANCE) Data Warehouse as a limited data set. The data used in this study come from the OCHIN Epic EHR (Devoe and Sears, 2013). This includes patient- and encounter-level information such as demographics, encounter details, vital signs, diagnoses, procedures, immunizations, laboratory tests, medications, and patient-reported outcomes. OCHIN is a nonprofit organization focused on equitable health care innovation and provides a centralized EHR system that results in the largest collection of community health data in the country. OCHIN members include Federally Qualified Health Centers, community health centers, rural health clinics, school-based clinics, behavioral health providers, dental clinics, public health departments, and HIV/AIDS care organizations.

The study was approved by the **[masked]** IRB. This study was conducted through a data use agreement with OCHIN.

### Patient Population

A limited dataset was curated by OCHIN. The initial dataset inclusion criteria was ambulatory or telehealth visits for adults 18 or older, between 2012 and 2019 so as to exclude visits occurring during the COVID-19 pandemic. Patients with ICD-9 or ICD-10 codes assessing mental disorders (F01-F99), diabetes (E08-E13), obesity (E65-E68), metabolic (E70-E88), and hypertensive (I10-I16) conditions were included in the dataset. Over 5 million patients met this initial criteria for the limited data set.

## Structured Measures

### Patient Health Questionnaire-9

The Patient Health Questionnaire is a well-established measure of depression symptomatology that was originally developed for assessing and monitoring depression severity using DSM-IV and ICD-10 criteria (PHQ-9; Kroenke & Spitzer, 2002). It has been tested extensively in racially and ethnically diverse populations (Huang et al., 2006), and in populations with medical comorbidity like diabetes (Nouwen et al., 2021; Udedi et al., 2019) and has been found to have similar performance across these populations when used as a screening instrument. The measure consists of 9 questions related to depression over the past 2 weeks. Scores range from 0 to 27, with higher endorsements indicating more symptoms and higher frequency of depression experiences. Conventional coding of the PHQ-9 scores are at the cut-points 5, 10, 15, and 20, representing mild, moderate, moderately severe, and severe levels of depression. At scores of 10 or greater, additional clinical attention is warranted, though this may not be specific to depressive disorders. Cut-points of 15 and higher suggest treatment intervention for depression is warranted. Item-level data were not available in the sample to calculate reliability; however, the PHQ-9 traditionally has been found to have excellent psychometric properties in a variety of samples, settings, and among people with different racial and ethnic backgrounds (Beard et al., 2016; Huang et al., 2006; Nouwen et al., 2021).

### Social Determinants of Health

The dataset contained several social determinants of health (SDH) screeners. Various measures were collected via EHR flowsheets, and currently only exist for OCHIN. OCHIN clinic sites used many different screening instruments, which have changed over time and are not currently standardized across health systems and clinic sites. Frequency of collection and use also changed over time. Approximately 22% of OCHIN active patients have been screened for at least one social need. Some SDOH data were recorded as part of demographics (e.g., federal poverty level).

### Data Analytic Plan

First, we pre-processed the data to create interpretable categories. The sample was restricted to any patient with an ICD code, a PHQ-9, or vitals available in the dataset. Data were cleaned to remove unlikely or outlier records. For example, a BMI exceeding 200 or less than 7, while possible, represents an extreme outlier and points to the possibility of human error in data entry or recording. We used a similar broad strokes cleaning approach for eliminating outlier values in self-report measures (e.g., negative values, fractional values). We then removed duplicate records and further restricted the sample to identify the first available encounter with a valid PHQ-9 score. If two or more valid scores were available on the same day, we randomly selected a record for inclusion in the models. Because providers switched from ICD-9 to ICD-10 at different points in the patient sample period, all ICD-9 and ICD-10 codes were recoded into more general codes. For example, patients with the codes for Major Depression, Dysthymia, Premenstrual Dysphoric Disorder, etc. were re-coded into the broader category of “Depressed”. Similarly, codes for Diabetes Mellitus and Type-2 Diabetes were collapsed into a single category of Diabetes.

Although the PHQ-9 does have a scoring algorithm that corresponds to diagnostic criteria (Kroenke & Spitzer, 2002), the item-level data needed to infer diagnoses was not available in the EHR dataset. We thus constructed 4 categories to represent different levels of severity for Patient-Indicated Depression: No Depression (< 4), Moderate Depression or Worse (PHQ > = 10), Severe Depression or Worse (PHQ > = 20), and Most Severe Depression (PHQ-9 = 26 or 27). The first 3 categories follow traditional PHQ-9 cutoffs; the fourth category of Most Severe was created to describe patients who would be extremely likely to meet diagnostic criteria for depression. In order to obtain a score of that magnitude, the patient would have had to endorse almost every item as occurring “Nearly every day”, and would have to, at a minimum, endorse the critical item of having thoughts of suicide or self-harm as occurring more than half of days or every day. Following feature selection, we used single imputation with random forest to handle missingness.

We used a supervised machine learning approach known as classification and regression trees (CARTs; Breiman et al., 2017). Relative to models like deep neural networks, CARTs are intuitive and relatively transparent algorithms. The tree starts with an initial node or root and then is split based on a decision point. These splits continue until the tree arrives at the leaf nodes or terminal nodes where no further splits can be found by the algorithm. Although more sophisticated implementations of tree-based algorithms exist (e.g., boosted trees, random forests), these can come at the expense of interpretability and explainability. For the purpose of the current study, to examine the impact of source of depression categorization, we found it more advantageous to use single CART models as these can be examined visually to understand how the categorization decision was determined. We additionally applied *SHapley Additive exPlanations* decision trees to visualize the most important features, or variables, used to predict the depression outcome.

To construct the classification trees, we first split the sample into 70% training data and 30% testing data to check for generalizability to an unseen sample. We planned to construct three classification trees: Tree 1 with depression defined as provider-indicated depression only from the EHR; Tree 2 with depression defined as patient-indicated depression at the Most Severe level only, and Tree 3 with patient-indicated depression as Most Severe and patient-provider-indicated depression from the EHR. We chose Most Severe for Tree 2 and Tree 3 as it resulted in the most certainty about patient-reported depressive symptoms. For example, interpretation of the PHQ-9 can be complicated by somatic conditions, and scores can be elevated without the patient necessarily meeting diagnostic criteria for depression due to nonspecific symptoms like sleep problems or fatigue and weight changes (Aagaard et al., 2023). Scores in the Most Severe range, a category we created to represent scores of 26 or 27 on PHQ-9, was expected to have high rates of true depression, even with concurrent chronic medical conditions (Kroenke et al., 2001).

For all three tree models, we tuned the hyperparameters within the training data using the *caret* package in R. The hyperparameters, including the minimum number of splits, splitting criteria (minimum 2 splits to ensure the tree split beyond the root), and class penalties, were iteratively adjusted to maximize the area under the ROC curve. A 10-fold cross-validation procedure was applied during training, such that in each iteration nine folds were used for model building and one fold was used for validation, with performance averaged across folds.

To evaluate our primary study aim, we compare the sensitivity and AUC values of the different models to establish the impact of provider-determined, patient-determined, or patient/provider concordant depression. We additionally report specificity, and F1 (i.e., the harmonic mean of positive predictive value and sensitivity), metrics for both cross-validation results and results with the holdout test set. A final version of this manuscript was spell-checked, grammar checked, and checked for APA style adherence with *Claude* Sonnet 4.6 (Anthropic, 2026) and exact prompts and results are available on the osf respository **[masked]**.

### Transparency and Openness

Raw data underlying this article were generated from multiple health systems across the OCHIN network; restrictions apply to the availability and re-release of data under organizational agreements. The study designed and analyses were not preregistered. However, code for tables and figures are available through an OSF repository **(masked)**. The data presented have not been previously published, in part or in whole, and there are no works currently in press works stemming from this same dataset.

## Results

### Sample characteristics

Characteristics of the sample are displayed in Table 1. A total of 644,387 cases remained following data pre-processing steps. Of those, 43% scored above the screening cutoff for Moderate Depression, 10% above the cutoff for Severe depression, and 1.3% above the Most Severe cutoff. Only 19% of patients had an ICD code for depression in the EHR. Very few (4.4%) patients with a low self-reported depression score were identified by providers as depressed; however, our Most Severe category of patient-indicated depression only had a provider-indicated ICD code for depression 44.5% of the time, suggesting a discordance between patient-indicated and provider-indicated depression.

### Differences in Classification Tree Performance by Depression Specification

We summarize the model metrics in Table 2. We found that sensitivity was low for all three models in the holdout test data (.28–.33). Sensitivity was highest for Tree model 1 (provider-indicated depression), followed by Tree model 3 (patient/provider concordant depression). Sensitivity ranges were similar in the cross-validation data that upsampled the depression class (.28–.40); however, it was Tree 3 that had the highest sensitivity value followed by Tree 1. Examining the positive predictive values and F1 in the test dataset, we concluded that it was likely that the low incidence rates of the Most Severe category contributed to challenges with generalizing model training to the holdout set. As a result, we trained and tested two additional models (Tree 2b and Tree 3b) that used the traditional PHQ-9 cutoff of 20 for “Severe”. These results, also presented in Table 2, indicated that Sensitivity in the holdout dataset improved noticeably (Tree 2b: .49; Tree 3b: .37), and performance on sensitivity exceeded Tree 1. Neither Tree 2b nor Tree 3b, exceeded the Tree 1, the provider-indicated depression model, in other metrics such as Specificity and F1, however.

### Differences in Feature Importance by Depression Specification

**Figure 1:**
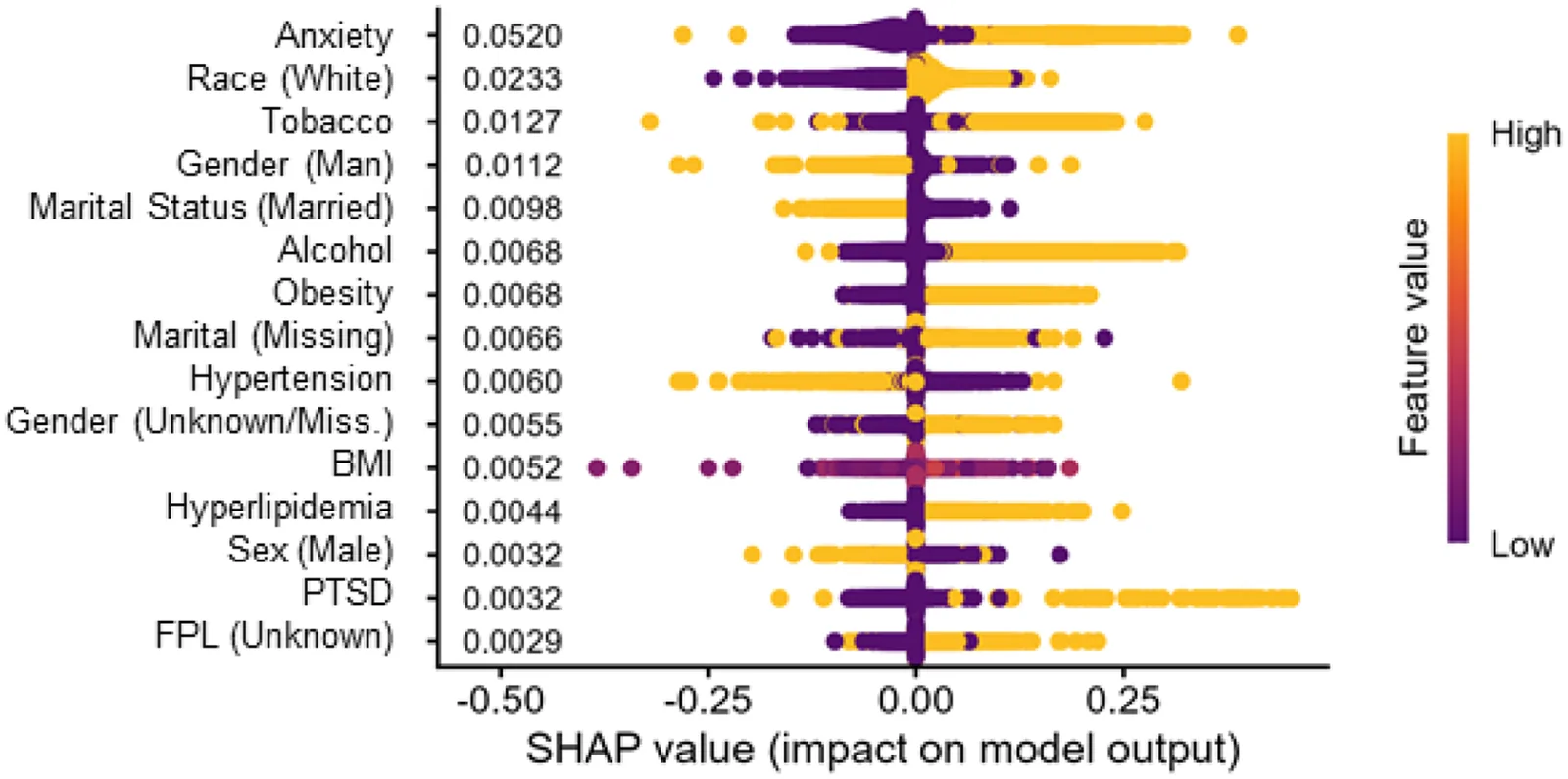
SHAP Beeswarm Plot of Feature Importance for Model 1 (Provider-Indicated Depression) *Note*. Each point represents an individual observation’s SHAP value. Color indicates the relative value of the feature with purple (darker) representing lower values and orange (lighter) representing higher values. Features are ordered by mean absolute SHAP value. FPL = Federal poverty level

Outcomes of SHAP analyses indicated that each model suggested a different set of values as being the most important for feature selection. (Fig. 1). The top 5 features for Tree 1 included having an anxiety diagnosis, having race listed as White, being unmarried, smoking, and female gender. Being younger, having an anxiety diagnosis, being unmarried, systolic and diastolic blood pressure were the top 5 features for patient-indicated depression. Lastly, younger age, having an anxiety diagnosis, being unmarried, not having hypertension, and being heterosexual were the top 5 features for Tree 3, the concordance model. Examining the beeswarm plots, most features had heterogeneous presentations that drove the model slightly more positive or slightly more negative. Interestingly, anxiety diagnosis was the only feature that consistently appeared in all three models but with slightly differing interpretations: in Tree 1, anxiety diagnosis was largely driving a positive depression diagnosis, in Tree 2, several patients with an anxiety diagnosis had driven the model away from a depression diagnosis, and in Tree 3 anxiety diagnosis largely drove the model towards a depression diagnosis, and absence of an anxiety diagnosis drove the model slightly away from depression.

## Discussion

The primary aim of this investigation was to determine what effect, if any, the choice of depression label source has on model prediction from a tree-based machine learning model. The model performance was fair overall with AUC values around .62 – .64 in both training and test data. Interestingly, the provider-indicated model had the best model metrics overall, although an alternative model for patient-indicated depression using the PHQ-9 cutoff of 20 instead of the “Most Severe” cutoff of 26 showed better sensitivity than the provider-indicated model. Our AUC findings are within range of other studies using EHR .55 − .94 but below the average of .78, among studies reporting AUC values. Granularity of data varied widely. For example, one study included structured and unstructured data in the form of mental health referrals (Kasthurirathne et al., 2019) to estimate postpartum depression; another study that trained a well-performing classifier, included demographic features, daily habits such as rhythm of meals and physical activity, and biomedical features (Nemesure et al., 2021). One meta-analysis found an AUC range of .70 − .79 among adequate-quality studies; however the best performing studies were treatment studies with access to genomic or neuroimaging data (Sajjadian et al., 2021). The heterogeneity of features as well the availability of features that reflect daily living habits that often correlate with depression (e.g., irregular meals), the authors may have had better predictive power than many other studies. Future studies focused on improving model performance using EHR record data may benefit from incorporating data from unstructured notes or using multimodal data for symptom detection (Aafjes-van Doorn et al., 2025).

Our findings align with this broader literature on variability in predictive performance, particularly when restricted to structured EHR data and they also point to a more fundamental issue that has received far less attention: the instability of ground truth in mental health datasets and the downstream consequences of label choice. Our study results highlight a critical gap in the current literature on predicting mental health outcomes with AI/ML models, revealing challenges both in model construction, which requires deeper understanding of mental health diagnostic processes and clinical workflows.

Ground truth is predominantly established via clinician-recorded diagnoses, clinician behaviors (e.g., prescription behavior), or biomarkers, without consideration of alternative label sources such as self-report measures of mental health symptoms that stem directly from the patient. Our findings show that label choice is not a *neutral* analytic choice. Ignoring patient-report as a source of diagnostic ground-truth stands in contrast to screening recommendations and measurement-based care guidelines that emphasize that the patient’s own report of symptoms is essential clinical information and a core part of high-quality depression assessment (Gaynes et al., 2008; Hong et al., 2021; Jackson & Machen, 2020; US Preventive Services Task Force et al., 2023). Underscoring this point are our findings that the clinical and demographic features emerging as important to the predictive model changed and shifted despite all records and features remaining identical across.

These patterns reflect a well-established principle of machine learning models – the model is always a reflection of the data used to produce it (Teno, 2023); however, our study demonstrates how hidden clinical workflow challenges have unique consequences in the mental health domain. There are longstanding structural challenges in implementing self-report symptom data collections into healthcare systems (Deane et al., 2025). Some prominent barriers to implementation of patient-reported symptom measures that physicians and even behavioral providers have reported are low clinical utility, lack of time, and beliefs that the data is unreliable due to patient factors like low-education or linguistic diversity (Dean et al., 2025; Keepers et al., 2023; Lewis et al., 2019). Implementation frameworks have been used to increase deployment of screening instruments and self-reported patient measures or to understand barriers to uptake (Gold et al., 2023; Wolff et al., 2025); however, our data also suggest that even if measure use is high, as we observed in our dataset, providers may not integrate results in the EHR. The problem of low rates of self-report data collection in hospital settings warrants further description and study in the context of AI and machine learning models if EHRs are to be used to develop and deploy predictive, diagnostic algorithms.

Our study also calls into question whether ML models that *do not* use patient-reported information can even be called “trustworthy”. There are several leading frameworks for trustworthy AI that encompasses guidelines for machine learning applications. The OECD framework, for example, emphasizes concepts of fairness, transparency, explainability, and robustness (OECD, 2021), whereas the European Union’s workgroup additionally emphasizes concepts of data governance and non-discrimination (*Ethics Guidelines for Trustworthy AI | Shaping Europe’s Digital Future*, 2019), indicating that trustworthiness goes beyond model performance. Indeed, human-computer and human-robot interaction researchers have adapted both performance and moral dimensions such as benevolence and ethics to trustworthiness dimensions (Mayer & Davis, 1999; Onnasch et al., 2025; Schlicker et al., 2025).

In mental healthcare where diagnosis depends on symptom information gathered through patient–provider interaction rather than biomarkers or independent clinical tests, we argue that trustworthy models should incorporate the patient’s “voice” or perspective; optimizing model performance alone is not sufficient. By applying a systematic framework for training models – establishing multiple ground truth labels, comparing model performance and predictions, and comparing explainability profiles – we demonstrated the trade-offs of using provider vs patient label sources and add to the literature calling for more routine bias assessment (Colacci et al., 2025). Models constructed without an aspect of patient-reported information risk distorting patient experience, amplifying existing provider-biases, and worsening the diagnostic practices they are meant to improve on (Chin et al., 2023; Osonuga et al., 2025). Such models may achieve acceptable performance metrics yet still fall short of the broader criteria for patient-centered trustworthy AI/ML algorithms.

### Limitations and Constraints on Generality

With regard to demographic diversity, our sample of over 600,000 patients were racially and ethnically diverse, but represented more Black, and fewer Asian patients than are present in the national census (U.S. Census Bureau, 2026). Our dataset was highly curated and response rates for patient-reported outcome measures like the PHQ-9 were high enough that a large sample of respondents could be obtained. Highly curated datasets like ours might not be representative of other healthcare systems in the United States, so care should be taken in generalizing to healthcare settings where screening instruments or documentation are inconsistent. Missing data was handled with single imputation to avoid the bias associated with listwise deletion; however, some measures had such low rates of data (e.g., specific SDOH items), that these measures were not included in the model. Other These omissions reflect a persistent challenge in representing patient-reports of their mental health symptom experiences in administrative EHR data.

### Future Directions

The goal of this investigation was to observe the impact of label choice on depression outcome rather than optimizing models for performance. Future studies should consider adopting our approach of systematically varying label choice in conjunction with testing a broad set of algorithms for performance improvements (e.g., random forest, deep neural networks, logistic regression).

### Clinical Implications

Our study demonstrates that clinicians and researchers invested in the use of AI for prognostic models should remain vigilant to evaluating the underlying assumptions of the label source or the “ground-truth” for the algorithm. Prior work has called attention to the potential for bias in AI/ML algorithms and highlighted models that replicate systemic societal inequities present in healthcare data (Chen et al., 2019; Obermeyer et al., 2019; Tay et al., 2022; Timmons et al., 2023; Wang et al., 2024). Our study offers an addendum to those findings by demonstrating the consequences of hidden assumptions about the ground truth for mental health diagnoses: if provider-coded depression diagnoses are the ground truth for model training, the model reflects a specific style of clinical practice that privileges the clinicians’ interpretation of patient symptoms over patient-provided information (Mullainathan & Obermeyer, 2022). If clinicians who prioritize and value integrative assessment practices or measurement-based care adopt ML systems built on diagnostic labels that exclude patient-reported symptom perspectives in the foundation of “ground truth”, they may unknowingly be creating a misalignment between the patient-centered values guiding their clinical work and the model-training practices shaping AI/ML development for mental healthcare applications.

Ground truth selection in mental health datasets is a deceptively complex problem. We encourage future researchers using ML and predictive algorithms to evaluate, at a minimum, how model performance and feature attribution change when patient-reported definitions of mental health conditions are weighted against providerreported definitions. Doing so enhances trustworthy AI/ML in mental healthcare and critically, can surface biases in seemingly well-performing models.

## Figures and Tables

**Figure 2:**
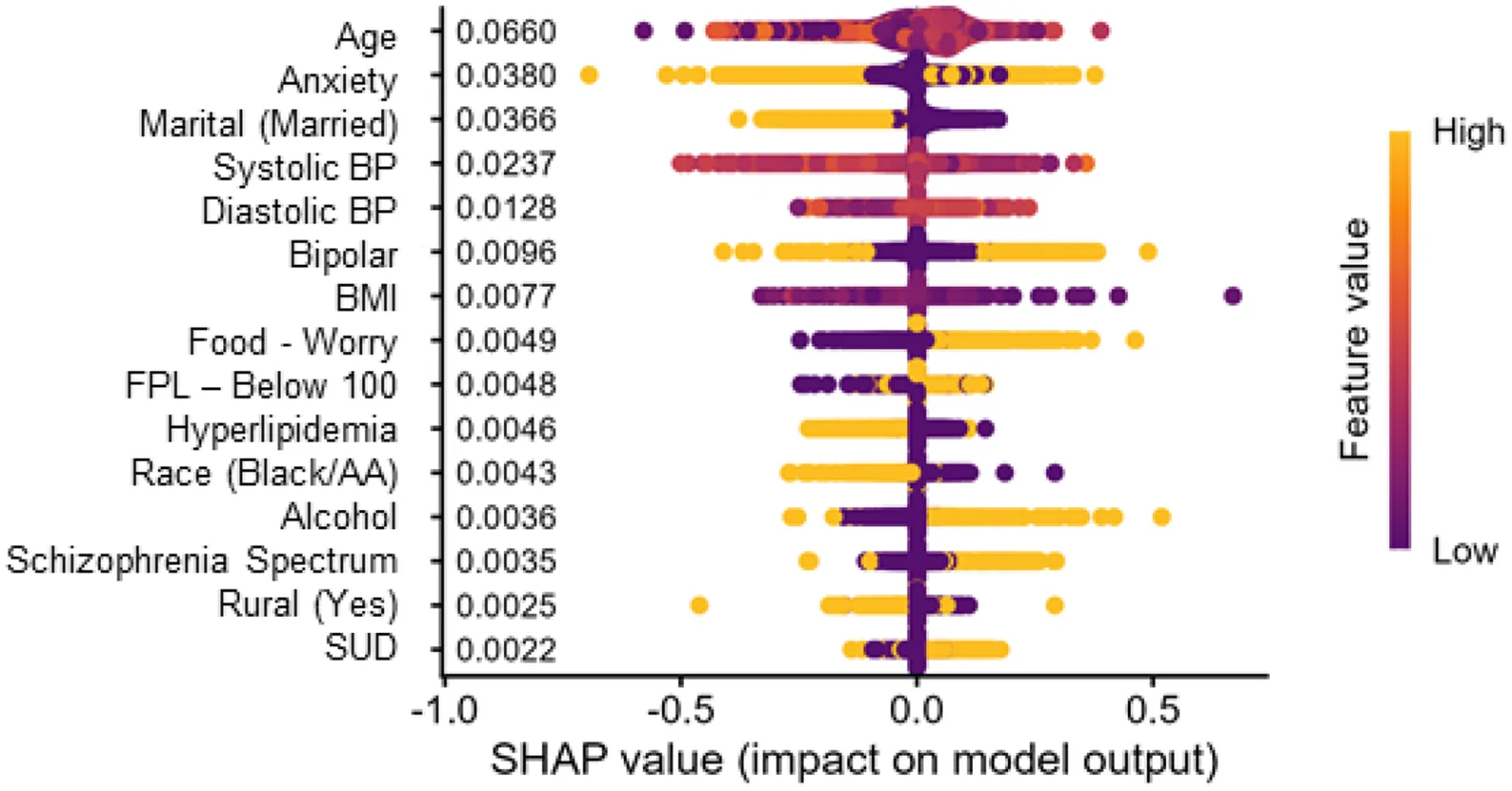
SHAP Beeswarm Plot of Feature Importance for Model 2 (Patient-Indicated Depression) *Note*. Each point represents an individual observation’s SHAP value. Color indicates the relative value of the feature with purple (darker) representing lower values and orange (lighter) representing higher values. Features are ordered by mean absolute SHAP value. FPL = Federal poverty level. Food–Worry = endorsed worry about food running out. AA = African American.

**Figure 3:**
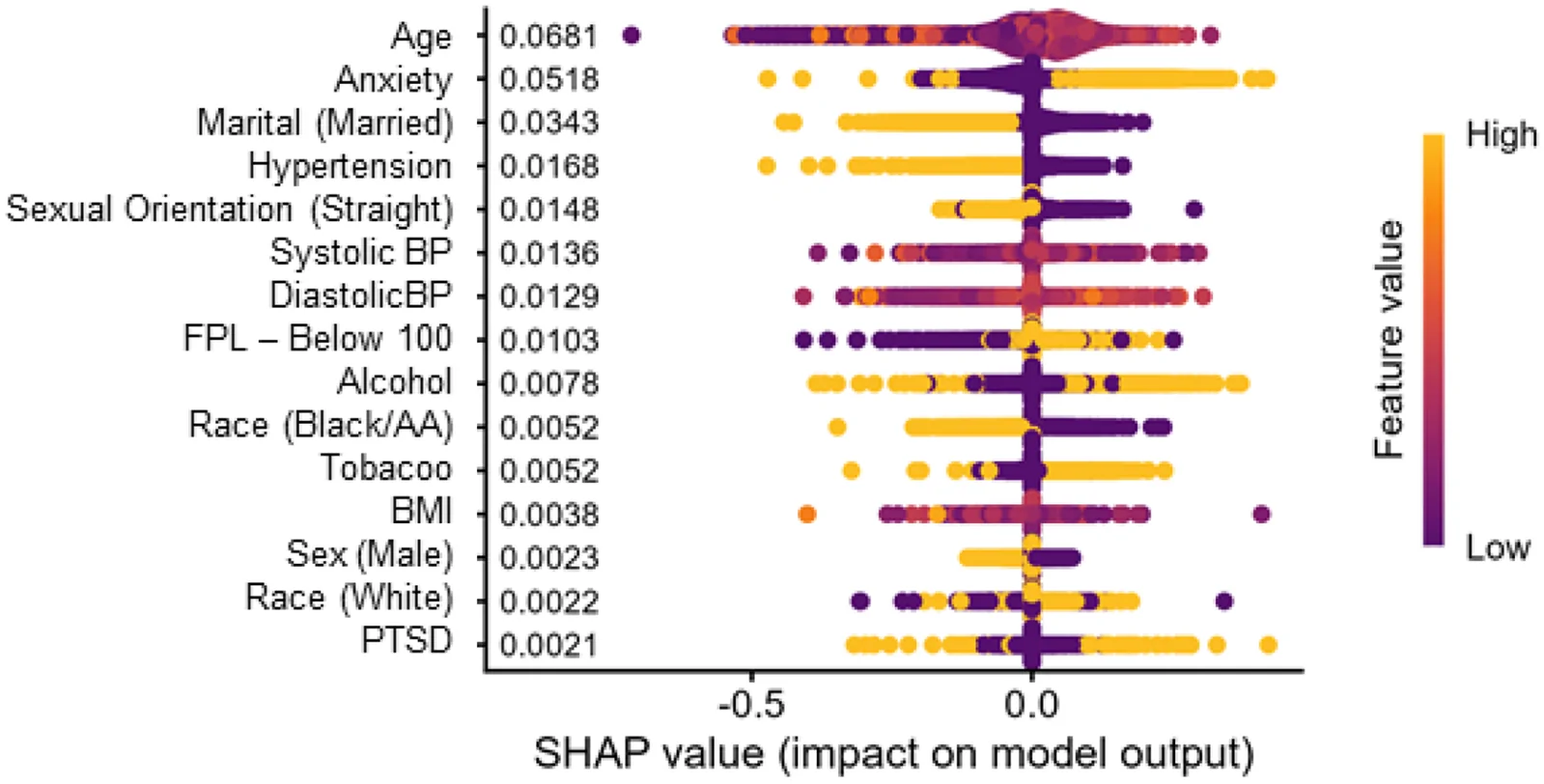
SHAP Beeswarm Plot of Feature Importance for Model 3 (Patient/Provider Concordant-Indicated Depression) *Note*. Each point represents an individual observation’s SHAP value. Color indicates the relative value of the feature with purple (darker) representing lower values and orange (lighter) representing higher values. Features are ordered by mean absolute SHAP value. FPL= Federal poverty level. AA = African American.

**Table 1:** Sample demographics and characteristics (N = 644,387)

Characteristic	Category	n (%)
**Gender**		
	Genderqueer	1,076 (0.2)
	Man	91,897 (19.9)
	Other	2,593 (0.6)
	Transgender	1,955 (0.4)
	Unknown / Missing	203,462 (44.1)
	Woman	160,616 (34.8)
**Race**		
	American Indian or Alaskan Native	4,729 (1.1)
	Asian	10,819 (2.5)
	Black or African American	80,332 (18.3)
	Multiple race	7,310 (1.7)
	Native Hawaiian or Other Pacific Islander	2,535 (0.6)
	Other	58 (0.0)
	Refuse to answer	12,409 (2.8)
	White	320,166 (73.0)
**Sex**		
	Female	289,783 (62.8)
	Male	171,599 (37.2)
	Sex No Information	<11 (0.0%)-
	Sex Unknown	>30 (0.0%)
**Sexual Orientation**		
	Bisexual	7,794 (1.7)
	Gay	4,648 (1.0)
	Lesbian	2,647 (0.6)
	Multiple sexual orientations	824 (0.2)
	Queer	739 (0.2)
	Something else	1,280 (0.3)
	Straight	210,723 (45.7)
	Unknown/ Missing	232,944 (50.5)
**Marital Status**		
	Divorced	22,866 (5.0)
	Domestic Partner	1,900 (0.4)
	Marital NI	186,057 (40.9)
	Married	74,531 (16.4)
	Other	1,065 (0.2)
	Separated	4,630 (1.0)
	Significant Other	7,550 (1.7)
	Single	147,610 (32.4)
	Widowed	9,088 (2.0)
**Federal Poverty Level**		
	FPL 101–150	56,466 (13.5)
	FPL 151–200	25,557 (6.1)
	FPL Below 100	275,225 (65.9)
	FPL Unknown	60,222 (14.4)
**Geography**		
	Rural = Yes	80,567 (17.5)
**PHQ Outcomes**		
	Above “Moderately Depressed” cutoff (10)	279,025 (43.3)
	Above “Severely Depressed” cutoff (20)	66,304 (10.3)
	Above “Most Severely Depressed” cutoff (26 or 27)	8,066 (1.3)
**Somatic Conditions**		
	Diabetes	66,818 (10.4)
	Obesity	49,492 (7.7)
	Hyperlipidemia	51,924 (8.1)
	Hypertension	87,764 (13.6)
**Psychiatric Diagnoses**		
	Depressed	121,933 (18.9)
	Bipolar	13,466 (2.1)
	Anxiety	105,406 (16.4)
	Alcohol	17,837 (2.8)
	Sleep	7,568 (1.2)
	SUD	23,822 (3.7)
	Tobacco	38,081 (5.9)
	Schizophrenia Spectrum	7,232 (1.1)
	Adjustment Disorders	4,591 (0.7)
	Eating Disorders	992 (0.2)
	Post-traumatic Stress Disorder	3,674 (0.6)

**Table 2.** Model performance metrics by cross-validation (CV) and test set

Model	10-fold Cross-Validation				Hold-out Test			
	AUC (95% CI)	Sensitivity(95% CI)	Specificity(95% CI)	AUC (95% CI)	Sensitivity	Specificity	PPV	F1
Tree 1: Provider-Indicated Depression	0.64 (0.63–0.64)	0.33 (0.32–0.34)	0.86 (0.86–0.87)	.63 (.63–.64)	0.33	0.86	0.37	0.35
Tree 2: Patient-Indicated Depression	0.62 (0.61–0.63)	0.28 (0.26–0.29)	0.86 (0.86–0.87)	.62 (.57–.66)	0.28	0.87	0.03	0.05
Tree 3: Patient-Provider Concordant Depression	0.64 (0.63–0.66)	0.40 (0.38–0.42)	0.82 (0.81–0.83)	.63 (.54–.73)	0.3	0.87	0.01	0.02

Note. Values in parentheses represent 95% confidence intervals (CIs).

AUC = area under the receiver operating characteristic curve; PPV = positive predictive value; F1 = F1-score. Cross-validation results are based on 10-fold CV; test set results are from the independent hold-out sample.

## Public Health Significance

Machine learning models developed from electronic health records are increasingly used to develop predictive models of mental health conditions like depression, yet most rely on provider-documented diagnoses. Through testing of multiple models with different label strategies for depression and including patient-reported outcomes, we demonstrate that depression label source meaningfully alters model performance and feature importance. Our results raise concerns about bias, interpretability, and generalizability in mental health prediction models.
